# A farm-level precision land management framework based on integer programming

**DOI:** 10.1371/journal.pone.0174680

**Published:** 2017-03-27

**Authors:** Qi Li, Guiping Hu, Talukder Zaki Jubery, Baskar Ganapathysubramanian

**Affiliations:** 1 Department of Industrial and Manufacturing Systems Engineering, Iowa State University, Ames, Iowa, United States of America; 2 Department of Mechanical Engineering, Iowa State University, Ames, Iowa, United States of America; University of Illinois, UNITED STATES

## Abstract

Farmland management involves several planning and decision making tasks including seed selection and irrigation management. A farm-level precision farmland management model based on mixed integer linear programming is proposed in this study. Optimal decisions are designed for pre-season planning of crops and irrigation water allocation. The model captures the effect of size and shape of decision scale as well as special irrigation patterns. The authors illustrate the model with a case study on a farm in the state of California in the U.S. and show the model can capture the impact of precision farm management on profitability. The results show that threefold increase of annual net profit for farmers could be achieved by carefully choosing irrigation and seed selection. Although farmers could increase profits by applying precision management to seed or irrigation alone, profit increase is more significant if farmers apply precision management on seed and irrigation simultaneously. The proposed model can also serve as a risk analysis tool for farmers facing seasonal irrigation water limits as well as a quantitative tool to explore the impact of precision agriculture.

## 1. Introduction

Farmland management under climate change and population growth is a pressing challenge that is becoming increasingly important due to food security considerations. The Institute for Operations Research and the Management Sciences (INFORMS), the leading professional association in analytics and operations research along with industrial interests, encouraged researchers to address the problem of feeding millions of people throughout the world who face hunger every day. There has been a growing body of literature on crop rotations at a regional scale [1, 2], land use patterns, and policy and environment issues on a farm scale [3]. Precision agriculture has attracted increasing attention in the community of farmland management. Over the years, the precision agriculture philosophy has enriched from simply "farming by soil" to a comprehensive system including irrigation planning, phenotypic selection, vehicle guidance systems, product quality and environmental management etc. [4–6]. As the demand for agricultural products increases, water and arable land become significant factors to improved agricultural production. Each year, farmers have to make decisions about what crops to plant given knowledge about the soil on their respective farms. Farmers need to select seed and plan for irrigation carefully to ensure maximum benefit from farming. Thus, crop planning and irrigation water management on a farm scale are imperative for improved agricultural productivity and sustainable development [7].

At the farm scale, farmers have a particularly strong incentive to optimize their water usage when the irrigation water price is high and the volume of available water is limited [8]. However, optimal usage of irrigation water resources requires efficient techniques and decision making support. There are mainly two approaches for this. On one hand, seed hybride selection is one method to improve water utilization. With the development of phenotype prediction and genotype selection, it is possible to utilize the high yield and drought resistant crop seeds. These new seed types give a farmer more flexibility to plant a variety of seeds on a farm, but also increase the difficulty for optimal pre-season seed planning. Alternatively, it is suggested that deficit irrigation is a more efficient method for water usage [9]. Deficit irrigation refers to the method that distributes a limited amount of irrigation water to satisfy essential water needs of plants [10]. Deficit irrigation could increase system benefits by saving water recourses, at the cost of individual benefits, by decreasing crop water allocation, especially during less critical periods of water demand. There are two major methods to implementing deficit irrigation for farmland. The first is to increase the interval between irrigation events. In other words, continue to irrigate with the same amount of water per irrigation as in the past but decrease the irrigation frequency (increase the number of days between irrigations). The second method of deficit irrigation is to irrigate at the same frequency as normal, but apply less water at each irrigation so that only a partial saturation level is achieved [11].

Mathematical programming has been widely used in farmland management, especially in irrigation management. Singh reviewed the literature in modeling, planning, and optimization of irrigation management with a focus on applications of different modeling techniques [12]. Sethi et al. developed a linear programming optimization model to find maximum annual net return for cropping and groundwater management [7]. The model was applied to a coastal river basin in India under different soil types, cropping patterns, and types of crops. Georgiou and Papamichail used simulated annealing and a gradient descent algorithm for reservoir and crop planning optimization [10]. Their method accounted for variable reservoir inflows and climate variability for crop planning. Wardlaw and Bhaktikul applied a genetic algorithm to optimize the delivery of water flows to minimize the distribution losses of an open race irrigation distribution system [13]. The major constraints in this study related to in-field soil moisture balances as well as canal capacities. Nagesh Kumar et al. used genetic algorithms for real-time reservoir operation management of multiple crops [14]. The study aimed to maximize the total yields from all crops considering reservoir inflow, the heterogeneous nature of soils, and crop response to the level of irrigation. Brown et al. used simulated annealing for on-farm irrigation scheduling considering seasonal water limits [8]. The objective was to maximize farm profit and was evaluated with a time-series simulation based on realistic plant growth models. Smout and Gorantiwar presented a water allocation linear programming model for optimizing the use of irrigation water to a medium irrigation scheme in India [15]. The model captured the deficit irrigation for each crop-soil-region combination. Yamout and El-Fadel developed a linear programming for setting policies for optimal water resources allocation on a regional scale [16]. Based on their study, the factors that greatly affect the water allocation scheme include profitability, public acceptability, and the effect of resources depletion.

It should be noted that most of existing studies focus on large scale management, such as, optimal irrigation and crop management on regional scale, and optimal scheduling for irrigation reservoir system. However, optimal on-farm level planning and irrigation scheduling remain a challenge from the research and practical perspectives [4]. For the studies focused on-farm level management, the granularity is typically a whole farm level, such as irrigation scheduling and crop rotation for the entire piece of land. Additional investigations are necessary to study the effect of the precision levels for on-farm management. In summary, majority of the literature focus on maximizing economic benefit, while maximizing yield and water use effectiveness were also adopted in several studies. Crop selection and irrigation management are among the main decisions to be made. Realistic constraints such as seasonal water limits, the heterogeneous nature of soils, and crop response to the level of irrigation applied are often considered. In this study, the proposed model aims at maximizing economic benefit by applying optimal decisions on crop selection and irrigation management. Seasonal water limits, soils features are considered. In addition, spatial structure and management scales are also considered in the proposed model to achieve a farm-level precision land management.

Corn, which is widely used for grain processing, food, beverages, livestock feed, and ethanol, takes up to one-third of cropland in the U.S. and is the nation's biggest crop economically. Corn receives the most irrigation water overall of American crops: approximately 19 billion cubic meters annually [17]. Eighty-seven percent of irrigated corn in the U.S. is grown in high or extremely high water stress regions such as the Great Plains and the Central Valley in California, and over half of it depends on groundwater from the over-exploited High Plains aquifer. Extreme weather events due to climate change affect the corn industry significantly. For instance, irrigation water costs have soared to $0.89/cubic meter in 2015 from approximately $0.11/cubic meter in 2014 in the Fresno-based Westlands Water District due to severe drought in California. The devastating Midwest drought of 2012 drove corn prices to a record of $315/metric ton. These facts provided motivations for this study.

Motivated by the gap between theoretical decision making challenges and the pressing application need in reality, the objective of this study is to develop a mixed integer linear programming model to provide decision support for customized precision farmland management. In the proposed model, decisions for pre-season seed selection and irrigation scheduling are made based on management properties such as types of soil, spatial structure, and management scales under a series of realistic constraints. Careful consideration was given to the model framework so that it could easily account for weather stochasticity in the future.

The remainder of the paper is organized as follows: in Section 2, the problem statement for the farm-level precision land management model is presented. The basic mixed integer linear programming model is introduced in Section 3. The authors illustrate the method with a case study in California and discuss the extension and modification of the basic model in Section 4. Finally, the paper concludes with a summary of research findings and potential research directions in Section 5.

## 2. Problem statement

Farmland management involves a sequence of planning and decision-making processes, the primary decision includes the scales and options of management. This paper focuses on solving two problems for farm-level precision land management. The first problem is to select the optimal crop management options within a customized management scale. The management options include seed type selection and irrigation frequency. The second problem is to choose the suitable management scale (size and shape) for these options. In other words, the model aims to assist the farmers to find the balance between precision level and management effort.

The "land unit" is defined as the minimum size over which management options are applied. The shape of a land unit is assumed to be square and the size of a land unit is informed by the measurement accuracy of soil types, agricultural working space, irrigation scale, and other physical limitations. Land unit could be viewed as the most precise block for a decision making level in farmland management. On the other hand, "decision unit" is defined as the farmer chosen scale for practical land management, which is a trade-off between convenience and precision. The size of decision unit could be any integer multiple of a land unit while the shape of a decision unit is a rectangle. A decision unit could be as small as one land unit or as big as the whole farmland. All the treatments and management options such as seed type selection and/or irrigation frequency setting in a decision unit are the same (among all land units in that decision unit). Based on these definitions, the problems could be a restatement of how to choose the scale of the decision unit and how to make optimal management option decisions within each decision unit. The hierarchical structure between land units and decision units make the proposed model flexible such that it can be extended to a farm that contains multiple disjoint pieces of land as well as to apply it to larger scales.

Several assumptions were made in the proposed model. It is assumed that the irrigate system already exists and it could apply different management options for each decision unit. It is also assumed that soil types will only affect the ability of holding water; they have the same nutrition levels [18]. The amount of water used in each irrigation is based on soil types, and the soils will achieve their saturated level after each irrigation [19]. It is assumed that irrigation will stop when the crops are dead. It should be noted that additional spatial constraints are included in the case study section to achieve a comprehensive analysis.

## 3. Model formulation

In this section, the mixed integer linear programming model for farmland management problem is introduced. The objective is to maximize the farmer's annual net profit when considering a specific farm.

### 3.1 Mathematical notations

The mathematical notations are summarized in Table 1.

**Table 1:** Notations for proposed model.

Subscripts		
*r*	1,2, …, *R*	irrigation frequency
*s*	1,2, …, *S*	seed type
*i*(*r*, *s*)	1,2, …, *I*	management option
*j*	1,2, …, *J*	land condition (soil types)
*m*	1,2, …, *M*	location of land unit in the horizontal axis
*n*	1,2, …, *N*	location of land unit in the vertical axis
*u*(*m*, *n*)	1,2, …, *U*	land unit (and its location)
*v*	1,2, …, *V*	decision unit
Binary decision Variables		
*x*_*iu*_	whether management option *i* is used in land unit *u*	
*y*_*ru*_	whether irrigation frequency option *r* is used in land unit *u*	
*z*_*su*_	whether seed type *s* is used in land unit *u*	
Parameters		
*A*	size of total farmland	
*B*_*v*_	set of land unit in decision unit *v*	
*E*	size of land unit	
*C*^*o*^	overhead cost (cash and non-cash)	
*C*^*w*^	unit cost for water	
$C_{ij}^{f}$	fixed cost of each irrigation for management option *i* used for land condition *j*	
$C_{ij}^{m}$	other farm operating cost for management option *i* used for land condition *j*	
*L*_*ju*_	land conditions *j* for land unit *u*	
*W*_*ij*_	amount of water needed for irrigation when management option *i* used for land condition *j*	
*Y*_*ij*_	unit maize yield when management option *i* used for land condition *j*	
*Y*	minimum yield requirement for the farmland	
*B*^*m*^	budget limit for other farming cost	
*B*^*w*^	budget limit for irrigation	
*R*^*b*^	unit revenue for selling biomass	
*P*	unit market corn price	
*W*^*l*^	irrigation water limitation per season	
*W*^*p*^	unit pre-irrigation water amount	
*Z*	objective value	
*α*	residues index	
*β*	sustainability factor	
*γ*	water use efficiency	

### 3.2 Objective function

The objective is to maximize the farmer's annual net profit, which is defined as the total revenues subtracted by total system costs during the farming process. The binary decision variable *x*_*iu*_ represents whether management option *i* is used in land unit *u*. The total revenues include revenue from selling crop grain as well as net revenue from selling the by-product crop residues. For example, corn stover, which is the residues after harvesting the corn grain, is an important feedstock for production of second generation biofuels [20]. Alpha (*α*) is the residues index that is defined as the mass ratio between crop grain and biomass residues. Beta (*β*) is the sustainability factor which is the percentage of biomass residue that has to be left in the field to sustain the soil nutrients. Evapotranspiration, also known as crop water use, is defined as the water removed from the soil by evaporation from the soil surface and transpiration by the plants. Evaporation can account for 20% to 30% of growing season evapotranspiration. Gamma (*γ*) is defined as the water use efficiency, the ratio between evapotranspiration and total purchased irrigation water. Table 2 summarizes the mathematical formulation of components in the objective function.

**Table 2:** Components in the objective function.

Component	Mathematical formulation
Crop sales revenue	*$\sum_{i=1}^{I}\sum_{j=1}^{J}\sum_{u=1}^{U}x_{iu}L_{ju}Y_{ij}EP$*
Residue sales revenue	*$\sum_{i=1}^{I}\sum_{j=1}^{J}\sum_{u=1}^{U}x_{iu}L_{ju}Y_{ij}E\alpha (1-\beta )R^{b}/(1-\alpha )$*
Other farming operating cost	*$\sum_{i=1}^{I}\sum_{j=1}^{J}\sum_{u=1}^{U}x_{iu}C_{ij}^{m}L_{ju}E$*
Water purchasing cost	*$\sum_{i=1}^{I}\sum_{j=1}^{J}\sum_{u=1}^{U}x_{iu}L_{ju}W_{ij}C^{w}/\gamma$*
Irrigation labor and equipment cost	*$\sum_{i=1}^{I}\sum_{j=1}^{J}\sum_{u=1}^{U}x_{iu}L_{ju}C_{ij}^{f}E$*

A variety of system costs have been considered in the model including labor costs, irrigation costs, machinery costs, seed costs, chemicals costs, cash overhead, and non-cash overhead. Cash overhead consists of various cash expenses during the year that are assigned to the whole farm such as insurance, office expenses, machinery maintenance, and field supervisors' salary. Non-cash overhead includes capital recovery cost (annual depreciation and interest costs) for equipment and other farm investments. In order to have a concise expression and focus on the impact of irrigation water management, several costs including labor costs, machinery costs, seed costs, and chemicals costs are lumped into a single cost called "other farm operating costs". Irrigation cost includes water purchasing cost and a fixed cost of labor and equipment. *C*^*o*^ represents the overhead cost per acre (cash and non-cash). The objective function is thus defined as follows.

$$\underset{x_{iu}}{\text{max}}Z=\sum_{i=1}^{I}\sum_{j=1}^{J}\sum_{u=1}^{U}x_{iu}L_{ju}\left(Y_{ij}EP+Y_{ij}E\alpha \left(1-\beta \right)R^{b}/\left(1-\alpha \right)\right)-\sum_{i=1}^{I}\sum_{j=1}^{J}\sum_{u=1}^{U}x_{iu}L_{ju}\left(C_{ij}^{m}E+C_{ij}^{f}E+W_{ij}C^{w}/\gamma \right)-C^{0}A$$

### 3.3 Constraints

The farming process requires upfront investment, which affects a farmer's cash flow. Farmers set up budget limits for certain cost categories. Constraint 1 ensures the total irrigation cost is below its budget. Constraint 2 ensures that other farm costs are below budget limit. No budget limit is set for overhead cost since it is independent from management decisions. For the consideration of food safety and a stable market, the government will encourage farmers to produce at least certain amount of crop in some cases. Similar total yield constraints are needed when there is a contract for a yield mandate. These situations are indicated in Constraint 3. Meanwhile, as a vulnerable and valuable resource, the amount of irrigation water is often limited in a growing season [8]. This irrigation water limitation is reflected in Constraint 4. Constraint 5 ensures that the management decisions of land units are the same within a certain decision unit. Constraint 6 ensures that the irrigation frequency decisions are uniform within a certain decision unit. Constraint 7 ensures that the seed type selection decisions are uniform within a certain decision unit. It is noteworthy that the decision unit for irrigation is not necessarily the same as the decision unit for seed type. Only one type of decision could be made for each land unit, as indicated in Constraints 8 and 9. Constraints 10 and 11 govern that comprehensive decisions should be chosen from the union feasible region for each individual decision. The binary nature of decision variables are defined in the Constraint 12.

$$\sum_{i=1}^{I}\sum_{j=1}^{J}\sum_{u=1}^{U}x_{iu}L_{ju}\left(C_{ij}^{f}E+W_{ij}C^{w}/\gamma \right)\leq B^{w}$$ (1)

$$\sum_{i=1}^{I}\sum_{j=1}^{J}\sum_{u=1}^{U}x_{iu}C_{ij}^{m}L_{ju}E\leq B^{m}$$ (2)

$$\sum_{i=1}^{I}\sum_{j=1}^{J}\sum_{u=1}^{U}x_{iu}L_{ju}Y_{ij}E\geq Y$$ (3)

$$\sum_{i=1}^{I}\sum_{j=1}^{J}\sum_{u=1}^{U}x_{iu}L_{ju}W_{ij}/\gamma +W^{p}A\leq W^{l}$$ (4)

$$x_{iu}=x_{iu^{\prime }}\;\forall u,u^{\prime }\in B_{v}\;$$ (5)

$$y_{ru}=y_{ru^{\prime }}\;\forall u,u^{\prime }\in B_{v}$$ (6)

$$z_{su}=z_{su^{\prime }}\;\forall u,u^{\prime }\in B_{v}$$ (7)

$$\sum_{r=1}^{R}y_{ru}=1\;\forall u$$ (8)

$$\sum_{s=1}^{s}z_{su}=1\;\forall u$$ (9)

$$x_{iu}\leq y_{ru}\;\forall u$$ (10)

$$x_{iu}\leq z_{su}\;\forall u$$ (11)

$$x_{iw},\;y_{rw},\;z_{su}\in \left\{0,1\right\}\;\forall i,r,s,u$$ (12)

## 4. Case study

In California, the total area planted for field corn was 210 436 hectares (520,000 acres) with the highest corn grain production occurring in Central Valley. Meanwhile, the overextended Central Valley aquifer is one of the most vulnerable water resources, which could create additional risks for the $65 billion-a-year corn industry [17]. As an irrigated summer crop, the amount of irrigation applied to California field corn will largely determine how much water is available to the crop. Thus, it is imperative to implement precision farm management in this area. In this section, a farm located in Yolo County, Central Valley, California, is selected to conduct a case study and illustrate the proposed model. The size of the land is 65.56 hectares (162 acres) and the shape of the land is square. Extensions of the basic model on different implementation conditions are also discussed.

### 4.1 Data source

**Fig 1:**
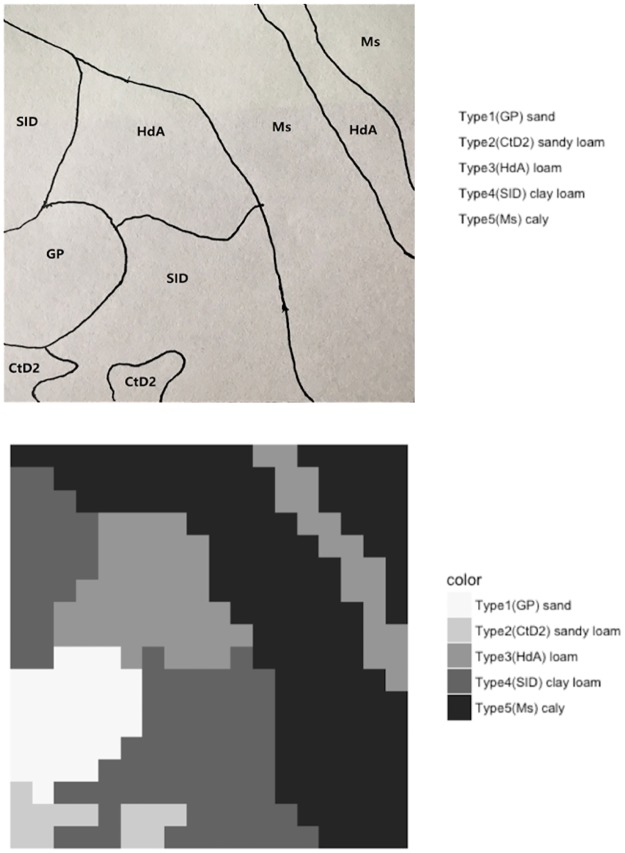
Schematic map (upper) and integrated map (lower) for soil types.

In the Central Valley, corn planting occurs from March through June and the time to mature is about 80 days to 130 days depending on the variety. Broadly, corn development can be divided into the vegetative stage that lasts through tassel and the reproduction stages that include silking, pollination, and grain filling. Since the plants don't consume much water in the early vegetative stage (first 4 weeks) and do not need much irrigation, this study only considers the reproduction stages which involves irrigations (approximately 15 weeks). A variety of soil textures are present in the farms used for field corn production. Sandy soils are preferred for early plantings because they warm rapidly in the spring. Heavier soils are productive, provided they are well drained and properly irrigated. The soil information up to 0.91 meters (36 inches) in depth is collected using the Web of Soil Survey. This information is used to define six integrated soil types (sand, loamy sand, sandy loam, loam, silt loam or clay loam, and clay) based on the Unified Soil Classification System (USCG). The water holding capacity of the soil types are adapted from literature [21, 22]. As shown in the upper part of Fig 1, there are five different types of soil in this farmland. Type 1 is the sand soil and Type 5 is the clay soil. This piece of land is divided into 324 land units and each land unit is a square with an acreage of 0.2 hectares (0.5 acres). If there are more than one soil type in a land unit, majority vote is applied to decide the soil type for that land unit, as shown in the lower part of Fig 1.

The Central California Irrigation District (CCID) is one of the largest irrigation districts in the Central Valley, serving over 1,600 farms across more than 57870 hectares of prime farmland. The price of irrigation water is volatile and varies significantly by location, water usage, and water type (well water or surface water). In this study, farmers use both well and surface water at an average price of $0.073/cubic meters ($90/acre-foot). Seasonal irrigation water limits are set when insufficient water is available due to weather conditions and government regulations. For example, the CCID set seasonal irrigation water limits to be 2664 cubic meters (2.16 acre-feet) in 2014 and 3700 cubic meters (3 acre-feet) in 2015. The baseline of total water available is set to be 3083 cubic meters (2.5 acre-feet) per season in the case study. Six irrigation frequencies are available for selection (every day, every week, every other week, every three weeks, every four weeks, and never). Irrigation cost and overhead cost information are based on estimates from the Natural Resources Conservation Service (NRCS) and University of California Cooperative Extension [23]. Currently, almost all corn grown in California is irrigated by surface irrigation. In this study, the surge irrigation system is used with a water use efficiency of 0.6, meaning that 40% of the purchased water is lost during transportation, irrigation, and soil penetration. A pre-irrigation of 822 cubic meters (8 acre-inches) is applied in March. Other farm operating costs are estimated as $1333/hectare ($358 for machinery, $91 for labor, and $884 for seed and chemicals), and these costs are uniformly applied [24].

Researchers from the University of California, Davis, reported a yield range from 12.54 to 18.81 metric ton per hectare with a minimum 1131 cubic meters survival water requirement for corn. In this analysis, twelve candidate grain corn seeds are created: three seeds for each of four major seeds types, including stringy, drought, smart, and extravagant. These seeds have different levels of drought resistance and have a yield range from 13.17 to 18.81 metric ton per hectare [25]. These seeds share the same time needed to mature with a total evapotranspiration of approximately 63.5 cm. The planting density is on average 83950 per hectare for each seed type. The average annual price received by U.S. corn producers from marketing years 2000 to 2015 is $141/metric ton, with a range from $71.65/metric ton to $271.26/metric ton according to the National Agricultural Statistical Service (NASS) of the U.S. Department of Agriculture. The baseline for corn market price in the case study is set at $141/metric ton. Corn stover could be used to serve as an abundant source of winter feed for cattle, and can also be used as the feedstock for biofuel production. The annual corn stover yield is estimated based on corn grain yield with a residue harvest index of 0.5, meaning 50% of the above ground biomass is grain and the amount of corn stover is the same as grain [26]. Papendick and Moldenhauer [27] showed that a 30% removal rate results in 93% soil cover after residue harvest. Thus, the sustainability factor (*β*) is set to be 0.3. It is assumed that the farm under consideration does not have a baler and therefore prefers to sell unharvested stover and let the buyer do harvesting. The unit revenue for selling unharvested cornstalks is $35 per metric ton [28]. All cost data have been adjusted for inflation to 2015 U.S. dollars.

### 4.2 Results for Model I

**Fig 2:**
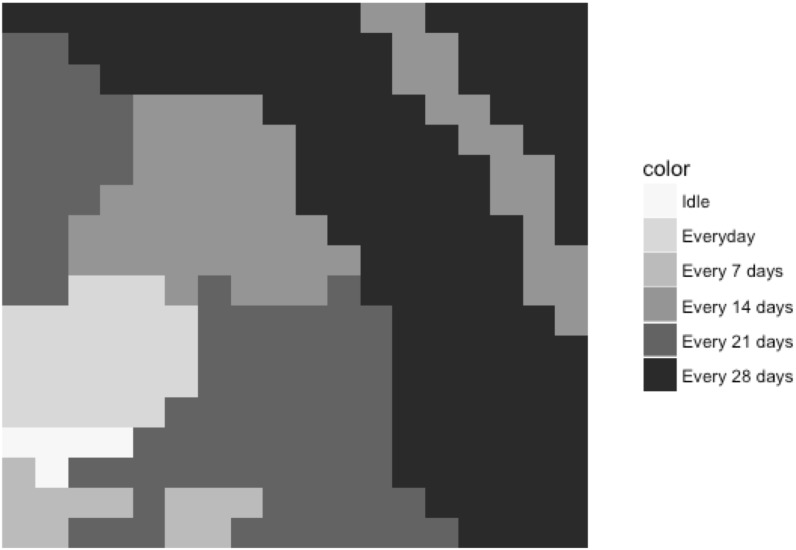
Irrigation decisions for basic model.

Model with Constraints 1 to 4, and Constraint 12 is defined as the Model I. The objective function and major constraints are consistent with the literature [7, 15]. In Model I, the size of a decision unit is set to be equal to the land units. Spatial structure and management scales are included in other models which will be defined later. Fig 2 shows that the managing option decisions are mainly chosen by the soil conditions; sandy land needs irrigation more frequently while clay land needs less irrigation. All decision unit chose the same seed type.

These results are consistent with a "farming by soil" philosophy [4]. Part of the sandy land is idle due to the total irrigation water amount limitation. The net profit for this 65.56 hectare of farmland is $29,615, which yields to an average profit of $451.85/hectare. In order to have a baseline for comparing with previous literature and different model settings, a baseline scenario is introduced. In the baseline scenario, the size of a decision unit is set to be the entire land (uniform decisions for the whole farmland). The model yields to an average profit of $113.22/hectare under this scenario. University of California Cooperative Extension reported an average profit range of $72.65/hectare to $135.91/hectare under same corn price with similar conditions [23]. The baseline scenario’s profit located at higher part of this range. The average profit of $113.22/hectare from baseline scenario will be used for comparison between difference models.

Comparing results from Model I with the baseline scenario, although the Model I increase the profit significantly, these results require the most precise level of management, for example, valves in the surge irrigation system need to be switched at each irrigation. Model I should be regarded as the practice with highest precision requirements, which will serve as the upper bound on profitability.

### 4.3 Risk analysis

It should be noted that selection of modeling parameters is critical for the analysis results. In reality, the parameters in the model can exhibit great uncertainty due to market fluctuations, and extreme weather events. Sensitivity analyses, which consider the influence of one parameter on the objective at a time by assuming other parameters as constant, have been adopted as a paradigm to evaluate uncertainties in the parameters and their influence [29]. The parameters under investigation include corn market price *P*, irrigation water price *C*^*w*^, other farm operating cost $C_{ij}^{m}$, overhead cost *C*^*o*^, water use efficiency *γ*, and seasonal water limit *W*^*l*^. The ranges of corn market price *P* and irrigation water price *C*^*w*^ are based on historical data, while the ranges of other parameters under investigation are ±25% of the base level.

**Fig 3:**
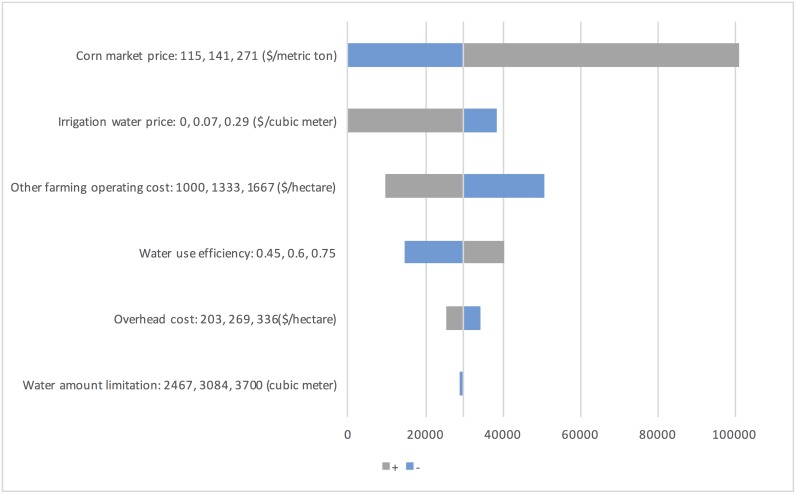
Sensitivity analysis of model parameters on annual net profit.

As shown in Fig 3, the parameters with largest impact on annual net profit are corn market price *P* and irrigation water price *C*^*w*^. The significant variation of these two parameters leads to high leverage for the annual net profit. Corn market price is influential because it is the key factor for gross income. The trigger price of corn market price for growing is $115.35/metric ton; corn market price lower than this point will lead to insufficient profit to cover farm costs. On the other hand, the termination price of irrigation water price is $0.28/cubic meters; irrigation water price that is higher than this point will make farming unprofitable. Extremely high irrigation water prices due to special weather events will affect net profit significantly. The annual net profit is also sensitive to other farm operating cost $C_{ij}^{m}$, and water use efficiency *γ*, which gives us insight about potential directions to increase annual net profit.

**Fig 4:**
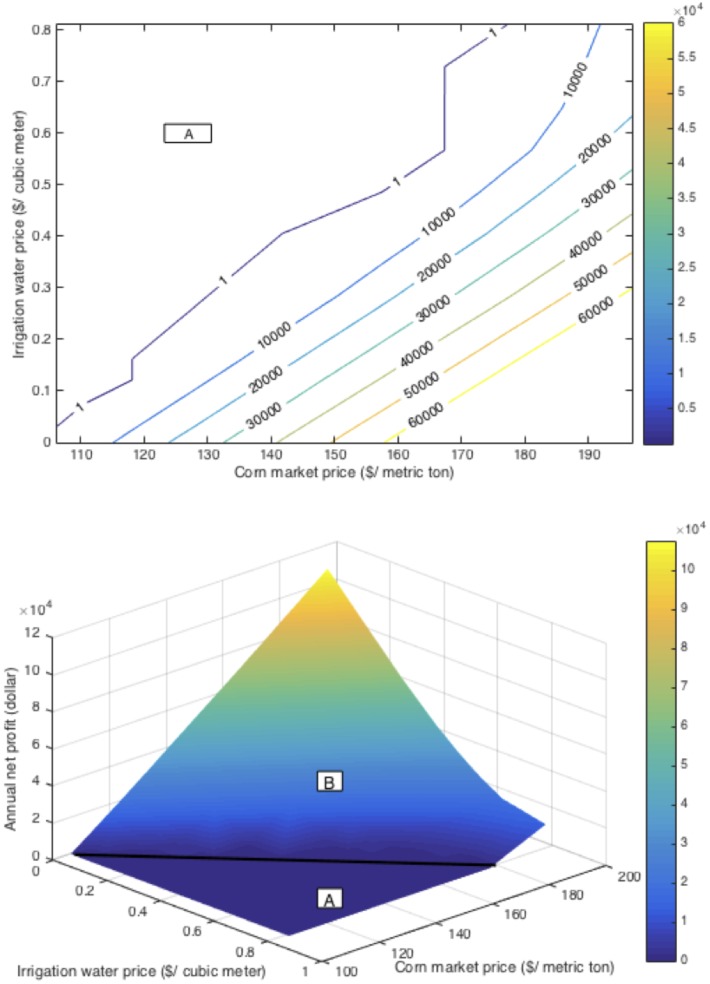
Contour plot (upper) and surface plot (lower) for profit region. Region A is the non-profitable region and Region B is the profitable region, the darkness indicates the profit level.

Due to market fluctuations and climate change, making decisions under specific scenarios became a widely concerning problem. In 2014 and 2015, several California irrigation districts could not provide irrigation water for Class II lands, which refers to soils with moderate limitations that reduce the choice of plants or require moderate conservation practices. Farmers of these lands have to pay for private well water at an auction price over $0.41/cubic meters, and the water suffers a loss factor related to the field's distance from the well source. A third of the Westlands district’s farmland (242811 hectares) were left unplanted in 2014 due to especially high irrigation water prices. The local government asked the farmers to conduct risk analysis before making decisions [30]. A risk analysis tool based on our basic model could be easily applied to these farmlands and give appropriate recommendations. The analysis shown already indicates that corn market prices and irrigation water prices are dominating parameters for annual net profit. The simultaneous change of corn market prices and irrigation water prices by assuming other parameters hold constant can give us insight about the profit region. As shown in Fig 4, Region A is the non-profitable region and Region B is the profitable region. If the speculated corn market price is relatively low and the irrigation water price is relatively high, farmers should change the crop type or leave the land idle to avoid further loss.

### 4.4 Discussion

In this section, some special cases are discussed to illustrate the flexibility of the proposed model. Additional constraints are included to make the model robust under different realistic assumptions such as spatial structure and management scales.

#### 4.4.1 Effect of size and shape of decision unit

Spatial relationship among land units are an essential part of the farmland model. In this section, the effect of size and shape of decision unit on annual profit are discussed. Even though precise farmland management will lead to more profit, it requires more management effort. The precision level and standardization level constitute a pair of tradeoffs. It is more realistic to make decisions on a larger scale and on a regular shape. In other words, each decision unit should contain multiple neighboring land units, and all land units in the same decision unit should share the same seed type and irrigation frequency. Three decision unit shape structures are investigated, namely square structure, row structure, and column structure. Meanwhile, several decision unit sizes are considered in order to find out the effect of scales.

Constraint 5 is added to the Model I, this new set of constraints ensure that the management decisions are uniform within a certain decision unit. This new constrained model will be referred to as Model II. Model I can be viewed as a special case of Model II with the scale of decision units equal to the land unit.

Sixteen scenarios are generated based on the size, shape, and number of decision units. The first scenario, which applies uniform decision for the whole farmland, is the baseline for comparison as introduced earlier. The gain ratio is defined as the annual net profit ratio between a certain scenario and the first scenario. Table 3 summarizes the annual net profit and gain ratio for each scenarios. As increasing the numbers of decision units (decreasing the size of decision units), the gain ratio is increased in general. These results indicate that detailed precision farmland management will bring high net profit. It also shows that the square structure is preferred because it has a higher gain ratio for the same size of decision unit and it has better flexibility. Table 3 also shows that the marginal benefit (of having more decision units) decreases. A highest gain ratio of 3.99 could be achieved by applying the philosophy of precision farm management.

**Table 3:** Effect of decision unit.

Scenario	Number of decision unit	Shape of decision unit	Net profit (dollar)	Gain ratio
1	1	Square	7423	1.00
2	2	Row	9954	1.34
3	2	Column	8003	1.08
4	3	Row	11009	1.48
5	3	Column	15157	2.04
6	4	Square	20672	2.78
7	6	Row	13765	1.85
8	6	Column	15157	2.04
9	9	Row	16104	2.17
10	9	Column	16516	2.22
11	9	Square	20816	2.80
12	18	Row	15918	2.14
13	18	Column	16516	2.22
14	36	Square	27148	3.66
15	81	Square	27915	3.76
16	324	Square	29615	3.99

#### 4.4.2 Special irrigation patterns

Up to this point, the model assumed that the decisions about seed type selection and irrigation frequency design are made simultaneously within each decision unit. However, due to the limitation of irrigation system, applying different irrigation frequencies to each decision unit may be cumbersome. Motivated by this practical limit, now the model allows different precision levels (size and shape of decision unit) between seed type selection and irrigation frequency design. Variables *y*_*ru*_ and *z*_*su*_ are included to indicate that each decision unit for seed type selection could have multiple irrigation frequencies and vice versa. Constraints 6 to 11 are added to Model II, and this new model is referred to as Model III. Model II can be viewed as a special case of Model III with a certain irrigation pattern.

Although Model III could capture any regular size and shape of decision unit in theory, three special irrigation patterns are investigated considering the irrigation system limitation, namely, Pattern 1: same irrigation frequency for each row (contains eighteen land units); Pattern 2: same irrigation frequency for each column (contains eighteen land units); and Pattern 3: same irrigation frequency for the whole farmland.

For each irrigation pattern, sixteen scenarios of seed type precision levels are investigated. These scenarios have the same definitions as in Model II. One dimension of precision management could be applied using Model III. On one hand, the authors want to find out the effect on annual profit by changing the precision level of seed type alone under certain irrigation patterns. On the other hand, the authors also want to investigate the effect on annual profit by changing the precision level of irrigation frequency alone under certain precision level of seed type selection. Under the same precision level of seed type selection, "relative gain for customized irrigation" (RGI) is defined as the net profit ratio between the highest profit (from Pattern 1, Pattern 2 or Model II) and profit for Pattern 3.

Table 4 summaries the annual net profit for three irrigation patterns under sixteen scenarios of seed type precision levels. As shown in the last column of Table 4, the RGI ranges between 2.00 to 2.42, which means that if the farmers have already decided the precision level of seed types selection, approximately 100% to 142% increase of net profit could be achieved by applying customized irrigation management. These increases are more significant under square decision units for seed type selection. On the other hand, if the farmers only allow precision management on seed type selection and use uniform irrigation frequency for the whole farmland (Pattern 3), the best gain ratio is 1.81. This result indicates that there is limited room for improving the net profit if farmers do not allow precision management on irrigation. When farmers allow some degree of precision management on irrigation (Pattern 1 and 2), the gain ratio will reach its upper bound at approximately 2.5.

**Table 4:** Effects of special irrigation patterns.

Scenario	Net profit (dollar)			RGI
	Pattern 1	Pattern 2	Pattern 3	
1	15529	16516	7423	2.22
2	15041	16516	7423	2.22
3	14484	16516	7423	2.22
4	14814	16516	8100	2.04
5	15529	16516	7423	2.22
6	15529	16516	8781	2.35
7	15403	16516	8100	2.04
8	14590	16250	7423	2.19
9	16104	16516	8184	2.02
10	14610	15411	7592	2.18
11	15878	16533	9681	2.15
12	16104	16403	8201	2.00
13	15894	16516	7522	2.20
14	17345	15882	11235	2.42
15	17718	18150	12111	2.30
16	19001	18265	13404	2.21
Best gain ratio	2.56	2.46	1.81	

To find out the quantitative relationships between the annual net profit with the number and shape of decision units under each irrigation pattern, regression analyses is conducted. Based on the hereinabove data analysis, logarithmic functions could be used to capture the effect of increasing the number of decision units, and a square structure has higher annual net profit under similar conditions. The following linear regression model is selected because it fits the data well and is easy to interpret.

$$P^{a}=\beta_{0}+\beta_{1}\text{ln}\left(n\right)+\beta_{2}\text{ln}\left(n\right)I\left(Shape=square\right)+\varepsilon$$ (13)

The response variable *P*^*a*^ is the annual net profit and the explanatory variable *n* is the number of decision units. ε is the random error that is not captured in the regression model, which is assumed to follow a normal distribution with mean zero and variance *σ*^2^. *I*(*) is the indicator function which takes value one when conditions are met and takes value zero when conditions are not met. *β*_0_ could be interpreted as the baseline of annual net profit when there is only one decision unit. *β*_1_ could be interpreted as the increment of annual net profit when the natural logarithm of the number of decision units increases by one. This increment will change to *β*_1_ + *β*_2_ when a square structure is selected. The best linear unbiased estimates and coefficient of determination (*R*^2^) are summarized in Table 5. These results show that choosing a square structure and having more decision units has a positive effect on the annual net profit. The effects of number and shape of decision unit are more significant when two dimensional precision management is applied. A logarithmic function could describe the accelerated decline of the effects from the number of decision units on the annual net profit quite well.

**Table 5:** Summary of regression analysis.

Parameters	Model II	Pattern 1	Pattern 2	Pattern 3
*β*_0_	10245.2	14707.7	16319.62	7416.06
*β*_1_	2233.4	344.3	65.59	184.06
*β*_2_	1747.2	363.8	239.25	864.08
*σ*	2583	502.6	275.7	275.3
*R*^2^	0.8738	0.8573	0.8131	0.9815

In summary, farmers could gain an additional 10%-80% net profit by employing precision management on seed type selection under certain irrigation patterns, and farmers could gain as much as an additional 142% net profit by working precision management on irrigation under certain seed type selection policy. One-dimensional precision management is relatively easier to implement but has a lower net profit. Precision management for irrigation appears to be more beneficial.

Besides confirming the dominant effect of crop prices and yields on net profit as stated in literature [23], this case study shows that irrigation water price, spatial structure, and management scales are also influential factors. The results from this case study show great economic potential of precision farmland management, and this recommendation is consistent with the literature [8].

#### 4.4.3 Potential for sustainable water usage

Although this study is mainly focused on economic analysis, it is important to take environmental issues into consideration. Water resources are limited and vulnerable, and corn is a thirsty plant. General strategies for coping with limited water include deficit irrigation of crops which can be stressed without significant loss of yield or quality, improving irrigation efficiency, improving crop genetics to develop varieties more tolerant to water stress, or planting other crops.

Reducing water amounts below what is required for corn will result in biomass reduction and grain yield reduction. What is more, the irrigation systems commonly used for corn in California do not allow close management of water stress. Thus, significant water savings can't be obtained by withholding water from the crop at present.

However, the method by which water is applied to the field could be improved. Strategies to maximize limited water include changes to irrigation management, design, or systems. Recall that in Model II and III, it is assumed that each land unit in a decision unit receives the same amount of water, which means some land units in a decision unit are over-irrigated and some water resources are wasted. Model I could eliminate this waste by having a land unit scale customized irrigation management.

Properly managed irrigation can apply a relatively uniform amount of water. However, application of high frequency may not be feasible with this system because of the labor input required for each irrigation. If farmers want to save water resources even further by applying deficit irrigation, new irrigation systems should be used such as sprinkler irrigation and traveling-gun irrigation. The proposed model could be easily modified to consider deficit irrigation. To illustrate this point, assume the irrigation technology could allow us to achieve at least partial saturation levels for a decision unit. Instead of assuming the corn cannot survive when it receives partial irrigation water, it is assumed that the yields of corn are depended by the saturation level. In other words, one more dimension of decision, the amount of irrigation water for each decision unit, are added in the model framework.

In summary, Model I is a special case of Model II with 324 decision units. Model II is a special case of Mode III with a certain irrigation pattern. These nested relationships indicate the flexibility of the proposed model.

## 5. Conclusions

In the study, a farm-level precision farmland management problem for pre-season seed type selection and irrigation water management is introduced. A mixed integer linear program is proposed with discussion on extensions and varieties of the basic model on different implementation conditions. Farmland in California serves as a case study to test the model's flexibility and economical optimality. The model gives qualitative descriptions and quantitative analysis for the management scale (number and shape of decision units). Special irrigation patterns are considered and the results show that the farmer's annual net profit could be significantly increased by applying one or two dimensional precision management decisions based on the proposed model. This model also serves as a decision making and risk analysis tool for farmers facing seasonal irrigation water limits and extreme drought conditions.

Note that this study is subject to a number of limitations. Firstly, the weather conditions such as temperature and rainfall are not considered in this model. These weather parameters affect the evaporation level of plants, pre-irrigation amount, and moisture level of the soil significantly. In addition, as discussed in the risk analysis, the parameters are not certain. Thus, a linear programming model with constant coefficients cannot fully describe the decision making environment [31]. Other modeling methods such as stochastic programming, dynamic programming, and robust optimization could be investigated [26]. In addition, multi-period models are needed for deficit irrigation design and invest new irrigation system.

The authors are working on a modified model which could take multi-period decisions of the seed hybrid and plant population selection, and amount of irrigation water, taking uncertain weather conditions and market price into consideration. This modified model would make the irrigation frequency and amount more flexible and precise. A stochastic program would be a natural fit to solve this problem; the first stage decision could be which type of plant seeds to grow while the other stage decisions could be the land management options such as irrigation amount for each irrigation.

The case study presented to illustrate and validate this optimization model only considers a certain piece of land. However, the shapes of farmland could affect the agricultural machinery paths and the homogeneous features of the soil could affect the shapes and sizes of decision units [32]. Motivated by finite element analysis, other future work includes develop models that allow different shapes and sizes of decision units in a piece of land. Last but not least, the proposed model could be used to evaluate other crops as well; and the interaction among plants such as plant population, leaf cover, and water competition could be stressed in future research.
